# Yunnan field mouse *Apodemus ilex* O. Thomas, 1922 (Rodentia, Muridae); a new species and genus for the Vietnamese fauna, first recorded from Son La Province

**DOI:** 10.3897/BDJ.14.e179239

**Published:** 2026-01-13

**Authors:** Alexander E Balakirev, Bui Xuan Phuong, Bui Thuy Trang, Viatcheslav V Rozhnov

**Affiliations:** 1 A.N.Severtsov's Institute of Ecology and Evolution of Russian Academy of Sciences, Moscow, Russia A.N.Severtsov's Institute of Ecology and Evolution of Russian Academy of Sciences Moscow Russia; 2 Joint Vietnam-Russia Tropical Science and Technology Research Centre, Hanoi, Vietnam Joint Vietnam-Russia Tropical Science and Technology Research Centre Hanoi Vietnam

**Keywords:** Biodiversity, Indochina, new species finding, occurrence

## Abstract

**Background:**

During fieldwork conducted in the Bac Yen District of Son La Province, near the Ta Xua Nature Reserve, a representative of a previously never registered genus and species of rodent in Vietnam, the Yunnan field mouse (*A. ilex* O. Thomas, 1922), was discovered.

**New information:**

This finding significantly expands our knowledge of the distribution of *Apodemus* species in northeastern Indochina. Morphological and genetic analysis of the specimen ruled out accidental introduction and established that the Vietnamese population is natural and belongs to the eastern phylogenetic branch of this species, which also inhabits the southern Yunnan-Guizhou Plateau, as well as the upper reaches of the Mekong, Hong Ha, and Da Rivers. Thus, the list of rodent species and genera in Vietnam has been expanded with a new species. Data on the species' geographic distribution across its range are also provided.

## Introduction

The genus *Apodemus* sensu lato is Palaearctic in distribution and contains 21 species, of which 9 occur in China and the Oriental region. *Apodemus* sensu lato is composed of two divergent groups (*Apodemus* sensu stricto and *Sylvaemus*). However, as many as 8 groups have been proposed out of which 4 to 5 may still be considered. Musser et al. (1996) divided *Apodemus* into three groups: *Apodemus* s.s. (*A. agrarius*, *A. chevrieri*, *A. speciosus*, *A. peninsulae*, *A. latronum*, *A. draco*, *A. semotus*, and *A. gurkha*); *Sylvaemus* (*A. sylvaticus*, *A. flavicollis*, *A. uralensis*, *A. mystacinus*, *A. fulvipectus*, *A. hermonensis*, *A. alpicola*, *A. arianus*, *A. hyrcanicus*, *A. ponticus*, *A. rusiges*, *and A. wardii*); and *Argenteus* (*A. argenteus*). *Alsomys* was allied with *Apodemus* s.s. whereas *A. argenteus* was considered distinct from the *Apodemus* and *Sylvaemus* groups. Musser and Carleton (2005) recognize four groups of *Apodemus* s.l., diverging from the traditional effort to classify the species under two or three subgenera. The *Apodemus* s.s. group consisting of 7 species (*A. agrarius*, *A. chevrieri*, *A. speciosus*, *A. peninsulae*, *A. latronum*, *A. draco*, *and A. semotus*) and distributed from Central Europe to Eastern Asia (most species have an Asian distribution) with endemic species in Taiwan (*A. semotus*) and Japan (*A. speciosus*) and one additional species *A. nigrus* described later (Ge et al. 2019). The *Sylvaemus* group consists of 11 species (*A. sylvaticus*, *A. flavicollis*, *A. uralensis*, *A. mystacinus*, *A. epimelas*, *A. alpicola*, *A. witherbyi*, *A. hyrcanicus*, *A. ponticus*, *A. rusiges*, and *A. pallipes*) that are distributed throughout most of Europe and the Middle East eastward to Central Nepal. The *Argenteus* group is composed of a single species *A. argenteus*, endemic to Japan, and the *Gurkha* group consists of the Nepalese endemic, *A. gurkha Pradhan 2018*.

Regarding the composition of the *Apodemus* group at the south-eastern border of its distribution, there are two species, namely the South China field mouse (*Apodemus draco* Barrett-Hamilton, 1900) and the Lantsang or Yunnan field mouse (*A. ilex* O. Thomas, 1922) recognised in the genus distributed here. They are two sibling species of mice with very similar morphology (Allen 1938, Ellerman and Morrison-Scott 1966, Liu et al. 2017) and once regarded as the same taxon, due to *A. ilex* for a rather long time considered a subspecies of *A. draco* (Xia 1984, Musser et al. 1996, Liu et al. 2017). It was originally described and named as a valid species by O. Thomas (Thomas 1922), with the type series collected at Mekong–Salween divide (28° 20' N), Yunnan (Allen 1940, Ellerman and Morrison-Scott 1951, Feng et al. 1986). Nevertheless, most authors (Allen 1940, Corbet 1978, Corbet and Hill 1992, Feng et al. 1986, Musser and Carleton 1993, Musser et al. 1996, Xia 1984) treated *ilex* as a synonym of *orestes* described by the same O. Thomas a little earlier (Thomas 1911, Thomas 1912) or *draco*, with the exception that Ellerman and Morrison-Scott (1951) considered it as a valid subspecies of another species, namely european *A. sylvaticus*.

There have been considerable controversies about the taxonomic status of *A. d. draco* and *A. d. orestes*. Corbet (1978) regarded *draco* as a separate species and treated *orestes* (which included *ilex*) as a valid subspecies of *A. draco*; however, Corbet and Hill 1992 extracted the former from *A. draco*, and treated it as a separate species. In contrast, Musser et al. (1996) combine *orestes* with the *draco* into a united taxon based on morphological comparisons of the samples from China and northern Burma. Recently, Jiang and Wang (2000) and Yang et al. (2002) morphologically examined the taxonomic status of *orestes* from Yunnan and Sichuan and suggested that the taxon is a valid species due to it having, e.g., significant differences in relative tail length from *draco*.

In more modern genetic analyses, Liu et al. (2004) first suggested, based on genetic peculiarities, that *ilex* should be recognized as a separate species and later provided substantial materials on its phylogeography and distribution (Liu et al. 2012). Over time, a series of studies on these species has investigated their distribution, taxonomic status and morphological characteristics (Allen 1938; Ellerman and Morrison-Scott 1966, Zimmermann 1962, Fan et al. 2010, Ge et al. 2019, Mezhzherin and Tereshchenko 2023), relative fatness and seasonal changes in digestive tract length (Du et al. 2001, Mu et al. 2012), eco-physiology (Li et al. 2009), karyotype (Chen et al. 1996; Motokawa et al. 2018) along with their molecular differentiation (Su et al. 1996, Liu et al. 2017, Ge et al. 2019) and reports on chigger mites' community composition inhabit these two sibling species in southwest China (Guo et al. 2023).

However, throughout the entire period of research, the genus *Apodemus* was considered strictly oriental, not distributed in Indochina. None of these species has ever been recorded south of 24 degrees north latitude. Specifically, according to the GBIF geographic information system, the southernmost point of *Apodemus ilex* is indicated as 25.68 N 100.1 E Cangshan, Dali, Yunnan, according to Kunming Institute of Zoology (KIZ) sample collection: KIZ: CS170131, KIZ: CS170051, KIZ: CS170004 genetic data, cytochrome b (*cyt b*) gene, partial cds mitochondrial sequence, May 2017). For *A. draco*, the southernmost point is determined to be Mu-cheng, Salween Drainage, China; Yunnan Province; near the border with Burma, coordinates 23.75N 99.166664E, according to samples of the American Museum of Natural History (AMNH) M-43752, M-43754, M-43744, M-43746 obtained 08-14.02.1917.

Hence, despite a significant amount of field research conducted in recent decades, representatives of this genus of mice have never been discovered in Vietnam or anywhere else in Indochina. *Apodemus* species are not listed in any mammal faunal surveys, and they are absent from taxonomic lists of Vietnamese mammals (Dang et al. 1994, Dang et al. 2008). However, during our recent fieldwork, we were lucky to find a representative of this group of mice in Vietnam, hundreds of kilometres south of the known species range. These findings and the point of the distribution of field mice near the southeastern boundary of the genus' range are the subject of this study.

## Materials and methods

**Figure 1.**
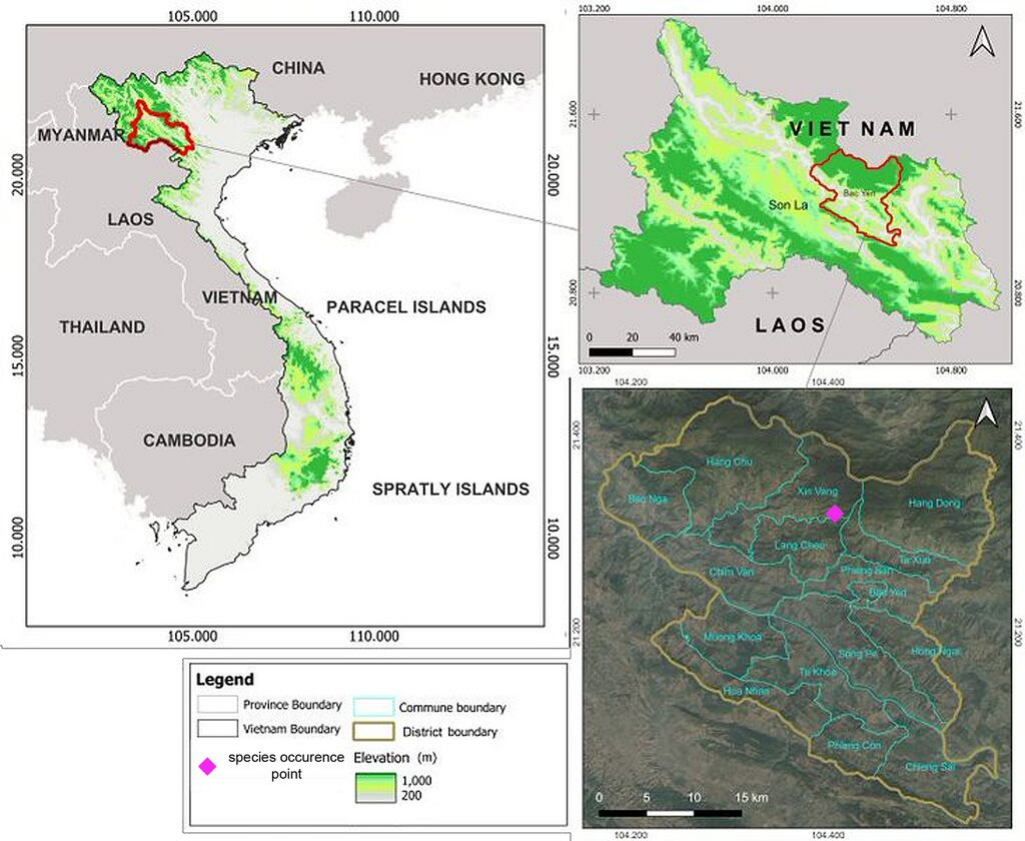
The study area and the location of the discovery point of the new species for Vietnam *Apodemus ilex* (Son La Province, Bac Yen District, Xin Vang commune, near village Suong Chong, Ta Xua Nature Reserve,21.319964N; 104.408068E, about 2000 m a.s.l.)

Our field surveys were conducted from November 21 to 30, 2023. The small terrestrial mammals were trapped using snap traps during one of the recent theriological expeditions organised by the Russian-Vietnamese Tropical Research and Technological Centre in Son La Province, Bac Yen District, within and in the vicinity of the Ta Xua Nature Reserve. Our goal was to analyse the diversity of small mammals in the area of the zoogeographic boundary between the eastern Indochina and south China biogeographical regions (Balakirev et al. 2025), which follows the Da River and crosses the study area. The point of animal capture is shown on the map in Fig. 1.

Species identification for small terrestrial mammal species was carried out in the field based on morphological characteristics (Francis 2008, Smith and Xie 2008). External measurement was performed directly in the field. The standard external body measurements (head and body length, tail length, hind-foot length, ear length) were taken. Animals were weighed with a compact digital balance. For confirmation of the record and further comparison with museum collections for clear taxonomical attribution, voucher specimens of each species obtained were taken by in-depth morphological and genetic analyses. For species for which precise identification based on external morphological characteristics was not feasible, as well as in questionable cases, species diagnostics were carried out later based on genetic data. All morphological samples were preserved in technical-grade 70% ethanol. Voucher specimens were catalogued and studied at the Zoological Museum of Moscow State University (ZMMU, Moscow, Russia). Small pieces of liver were stored in 96% molecular-grade ethanol and used for the DNA extraction and for genetic analyses.

Total genomic DNA was extracted using a routine phenol/chloroform/proteinase K protocol (Kocher et al. 1989, Sambrook et al. 1989). Each individual was genotyped using the *Cyt b* (1140 bp positions) and compared with all homologous sequences available in GenBank using the DNA BLAST procedure. Universal routine PCR protocols were used to amplify mtDNA fragments as follows: initial denaturation for 1 min 30 s at 95 °C, denaturation for 30 s at 95 °C, annealing for 1 min at 52 °C, and elongation for 45s at 72 °C, followed by terminal elongation for 3min at 72 °C. The PCR reaction was performed in a 25μl volume that contained 2.5–3mL of 10х standard PCR buffer, 50mM of each dNTP, 2mM MgCl_2_, 10pmol of each primer, 1 unit of Taq DNA polymerase, and 20–50ng of total DNA template per tube. The *Cyt b* gene was amplified directly using primer pair L14724 (5’-CGAAGCTTGATATGAAAAACCTCGTTG-3’; Irwin et al. 1991) and H15915 (5’-GGAATTCATCTCTCCGGTTTACAAGAC-3’; Kocher et al. 1989). The nucleotide sequence obtained was submitted to GeneBank and deposited under the ID PX565184.

To visualize the position of the new specimen in the species structure, a phylogenetic tree was constructed in MEGA using the Minimal Evolution (ME) method.

## Taxon treatments

### Apodemus (Apodemus) ilex

O. Thomas, 1922

3A428AF0-6AD4-5D14-80B3-AB3242471CB1

#### Materials

- **Type status:** Other material. **Occurrence:** occurrenceID: 82DEE185-6CDF-5BD6-A9EF-92B8BAFD9117; **Location:** higherGeography: Asia; country: China; countryCode: CH; verbatimLocality: Yulong Snow Mountain, Lijiang, Yunnan; decimalLatitude: 27.2000000; decimalLongitude: 100.2400000; **Identification:** identificationReferences: Song et al. 2021; identificationRemarks: getotyped
- **Type status:** Other material. **Occurrence:** occurrenceID: 85F53B25-F5BC-5B54-8855-D787C433AECF; **Location:** higherGeography: Asia; country: China; countryCode: CH; verbatimLocality: Zhiziluo, Fugong, Yunnan; decimalLatitude: 26.6000000; decimalLongitude: 99.0000000; **Identification:** identificationReferences: Song et al. 2021; identificationRemarks: getotyped
- **Type status:** Other material. **Occurrence:** occurrenceID: 64B91FF0-62C8-5476-AD51-7031E5F8B4E8; **Location:** higherGeography: Asia; country: China; countryCode: CH; verbatimLocality: Dandanglika, Dulongjiang, Yunnan; decimalLatitude: 28.1100000; decimalLongitude: 98.1800000; **Identification:** identificationReferences: Song et al. 2021; identificationRemarks: getotyped
- **Type status:** Other material. **Occurrence:** occurrenceID: B23C7ABB-F4B3-57A4-B89C-864220596F5B; **Location:** higherGeography: Asia; country: China; countryCode: CH; verbatimLocality: Cangshan, Dali, Yunnan; decimalLatitude: 25.6800000; decimalLongitude: 100.1000000; **Identification:** identificationReferences: Song et al. 2021; identificationRemarks: getotyped
- **Type status:** Other material. **Occurrence:** occurrenceID: DA75D392-111B-5FB6-85CC-B84B24C6F5F0; **Location:** higherGeography: Asia; country: China; countryCode: CH; verbatimLocality: Lushui, Yunnan; decimalLatitude: 25.9600000; decimalLongitude: 98.9600000; **Identification:** identificationReferences: Song et al. 2021; identificationRemarks: getotyped
- **Type status:** Other material. **Occurrence:** occurrenceID: 122A9DE5-6E9A-5A4F-B3C0-1B090E49AFA2; **Location:** higherGeography: Asia; country: China; countryCode: CH; verbatimLocality: Longma Shan, Yunlong, Yunnan; decimalLatitude: 26.2100000; decimalLongitude: 99.2200000; **Identification:** identificationReferences: Song et al. 2021; identificationRemarks: getotyped
- **Type status:** Other material. **Occurrence:** occurrenceID: 6943B142-993D-5C09-B7E1-C6EB1C5B55F8; **Location:** higherGeography: Asia; country: China; countryCode: CH; verbatimLocality: Zhiziluo, Fugong, Yunnan; decimalLatitude: 26.5900000; decimalLongitude: 99.0200000; **Identification:** identificationReferences: Song et al. 2021; identificationRemarks: getotyped
- **Type status:** Other material. **Occurrence:** occurrenceID: AE10B015-79C3-588F-BBE1-9183D46F6BB2; **Location:** higherGeography: Asia; country: China; countryCode: CH; verbatimLocality: Laojun Shan, Lijiang, Yunnan; decimalLatitude: 26.8500000; decimalLongitude: 99.6200000; **Identification:** identificationReferences: Song et al. 2021; identificationRemarks: getotyped
- **Type status:** Other material. **Occurrence:** occurrenceID: 7EC24DCB-E5EA-5BD9-9F72-F56B03FC5256; **Location:** higherGeography: Asia; country: China; countryCode: CH; verbatimLocality: Yaping, Fugong, Yunnan; decimalLatitude: 27.2100000; decimalLongitude: 98.7000000; **Identification:** identificationReferences: Song et al. 2021; identificationRemarks: getotyped
- **Type status:** Other material. **Occurrence:** occurrenceID: C2BA8601-DD7A-5C62-B108-43A198113881; **Location:** higherGeography: Asia; country: China; countryCode: CH; verbatimLocality: Haba Snow Mountain, Shangri-la, Yunnan; decimalLatitude: 27.3500000; decimalLongitude: 100.0900000; **Identification:** identificationReferences: Song et al. 2021; identificationRemarks: getotyped
- **Type status:** Other material. **Occurrence:** occurrenceID: A338792E-D9FC-576F-A9E3-DA265CAAC266; **Location:** higherGeography: Asia; country: China; countryCode: CH; verbatimLocality: Kangpu, Weixi, Yunnan; decimalLatitude: 27.6300000; decimalLongitude: 99.1200000; **Identification:** identificationReferences: Song et al. 2021; identificationRemarks: getotyped
- **Type status:** Other material. **Occurrence:** occurrenceID: DF24E05D-F7E9-583E-B296-022EA48F4EE3; **Location:** higherGeography: Asia; country: China; countryCode: CH; verbatimLocality: Shika Snow Mountain, Shangri-la, Yunnan; decimalLatitude: 27.7800000; decimalLongitude: 99.6000000; **Identification:** identificationReferences: Song et al. 2021; identificationRemarks: getotyped
- **Type status:** Other material. **Occurrence:** occurrenceID: 29D19631-B2F1-5B44-B32C-BFC18ABEA453; **Location:** higherGeography: Asia; country: China; countryCode: CH; verbatimLocality: Shika Snow Mountain, Shangri-la, Yunnan; decimalLatitude: 27.7900000; decimalLongitude: 99.5900000; **Identification:** identificationReferences: Song et al. 2021; identificationRemarks: getotyped
- **Type status:** Other material. **Occurrence:** occurrenceID: 8833597A-FAC6-549F-95A2-65E49AC9CA7C; **Location:** higherGeography: Asia; country: China; countryCode: CH; verbatimLocality: Bingzhongluo, Gongshan, Yunnan; decimalLatitude: 27.9500000; decimalLongitude: 98.5000000; **Identification:** identificationReferences: Song et al. 2021; identificationRemarks: getotyped
- **Type status:** Other material. **Occurrence:** occurrenceID: 5E2E8F82-C5E5-5069-BEC9-CFE294772BAA; **Location:** higherGeography: Asia; country: China; countryCode: CH; verbatimLocality: Gezan, Shangri-la, Yunnan; decimalLatitude: 28.1400000; decimalLongitude: 99.9000000; **Identification:** identificationReferences: Song et al. 2021; identificationRemarks: getotyped
- **Type status:** Other material. **Occurrence:** occurrenceID: 3D4C8688-1D8A-51DA-B18E-52BB356EA512; **Location:** higherGeography: Asia; country: China; countryCode: CH; verbatimLocality: Meili Snow Mountain, Deqin, Yunnan; decimalLatitude: 28.2600000; decimalLongitude: 98.7200000; **Identification:** identificationReferences: Song et al. 2021; identificationRemarks: getotyped
- **Type status:** Other material. **Occurrence:** occurrenceID: 06AE2AA8-3386-5FC3-AB39-FC702B9ECA4B; **Location:** higherGeography: Asia; country: China; countryCode: CH; verbatimLocality: Meili Snow Mountain, Deqin, Yunnan; decimalLatitude: 28.2800000; decimalLongitude: 98.7300000; **Identification:** identificationReferences: Song et al. 2021; identificationRemarks: getotyped
- **Type status:** Other material. **Occurrence:** occurrenceID: 8426D505-CDC1-5E4A-936F-C919DC757E57; **Location:** higherGeography: Asia; country: China; countryCode: CH; verbatimLocality: Meili Snow Mountain, Deqin, Yunnan; decimalLatitude: 28.2900000; decimalLongitude: 98.7300000; **Identification:** identificationReferences: Song et al. 2021; identificationRemarks: getotyped
- **Type status:** Other material. **Occurrence:** occurrenceID: D055252A-10BA-504F-902D-A4C1EC1D6948; **Location:** higherGeography: Asia; country: China; countryCode: CH; verbatimLocality: Baima Snow Mountain, Deqin, Yunnan; decimalLatitude: 28.3400000; decimalLongitude: 99.0300000; **Identification:** identificationReferences: Song et al. 2021; identificationRemarks: getotyped
- **Type status:** Other material. **Occurrence:** occurrenceID: 2EC02BAA-E9CC-50DE-BF8B-E38D26BAADD7; **Location:** higherGeography: Asia; country: China; countryCode: CH; verbatimLocality: Yubeng,Mt. Meili, YN; decimalLatitude: 28.3740000; decimalLongitude: 98.8150000; **Identification:** identificationReferences: Liu et al., 2012, 2018; identificationRemarks: getotyped
- **Type status:** Other material. **Occurrence:** occurrenceID: 2FC73391-A735-5740-AD19-C16BAC40269A; **Location:** higherGeography: Asia; country: China; countryCode: CH; verbatimLocality: Ciguzuzu field, Mt. Haba, YN; decimalLatitude: 27.3170000; decimalLongitude: 100.1090000; **Identification:** identificationReferences: Liu et al., 2012, 2018; identificationRemarks: getotyped
- **Type status:** Other material. **Occurrence:** occurrenceID: 2F311ACF-15A2-5482-A41C-618F277B7385; **Location:** higherGeography: Asia; country: China; countryCode: CH; verbatimLocality: Qiaotou, Mt. Haba, YN; decimalLatitude: 27.2910000; decimalLongitude: 100.1000000; **Identification:** identificationReferences: Liu et al., 2012, 2018; identificationRemarks: getotyped
- **Type status:** Other material. **Occurrence:** occurrenceID: 1E67792A-E56D-5854-BB17-2558414581FD; **Location:** higherGeography: Asia; country: China; countryCode: CH; verbatimLocality: Dajianshanping, Mt. Haba, YN; decimalLatitude: 27.3810000; decimalLongitude: 100.0900000; **Identification:** identificationReferences: Liu et al., 2012, 2018; identificationRemarks: getotyped
- **Type status:** Other material. **Occurrence:** occurrenceID: 8D5B2309-B160-5AC5-9F3D-18E1FE4211C4; **Location:** higherGeography: Asia; country: China; countryCode: CH; verbatimLocality: Daojiaoping, Mt. Haba, YN; decimalLatitude: 27.3550000; decimalLongitude: 100.0900000; **Identification:** identificationReferences: Liu et al., 2012, 2018; identificationRemarks: getotyped
- **Type status:** Other material. **Occurrence:** occurrenceID: 88E24D36-0514-59E3-81AF-DFC4317B944C; **Location:** higherGeography: Asia; country: China; countryCode: CH; verbatimLocality: Edi, Mt. Haba, YN; decimalLatitude: 27.4000000; decimalLongitude: 100.1200000; **Identification:** identificationReferences: Liu et al., 2012, 2018; identificationRemarks: getotyped
- **Type status:** Other material. **Occurrence:** occurrenceID: BAEA9EAF-34CD-5F41-9422-6907EE081565; **Location:** higherGeography: Asia; country: China; countryCode: CH; verbatimLocality: Daju, Mt. Yulong, YN; decimalLatitude: 27.2980000; decimalLongitude: 100.2500000; **Identification:** identificationReferences: Liu et al., 2012, 2018; identificationRemarks: getotyped
- **Type status:** Other material. **Occurrence:** occurrenceID: 54373FE7-E2AD-5A84-B4CB-3BD6A77FAE4F; **Location:** higherGeography: Asia; country: China; countryCode: CH; verbatimLocality: Maoniupinig, Mt. Yulong, YN; decimalLatitude: 27.1630000; decimalLongitude: 100.2480000; **Identification:** identificationReferences: Liu et al., 2012, 2018; identificationRemarks: getotyped
- **Type status:** Other material. **Occurrence:** occurrenceID: 6619A063-24FA-579E-A775-4A26E5C4F55A; **Location:** higherGeography: Asia; country: China; countryCode: CH; verbatimLocality: Ganhaizi, Mt. Yulong, YN; decimalLatitude: 27.1070000; decimalLongitude: 100.2580000; **Identification:** identificationReferences: Liu et al., 2012, 2018; identificationRemarks: getotyped
- **Type status:** Other material. **Occurrence:** occurrenceID: 62FB9BE3-6801-5705-BEAE-390E2701BDAF; **Location:** higherGeography: Asia; country: China; countryCode: CH; verbatimLocality: Yunshanping, Mt. Yulong, YN; decimalLatitude: 27.1140000; decimalLongitude: 100.2190000; **Identification:** identificationReferences: Liu et al., 2012, 2018; identificationRemarks: getotyped
- **Type status:** Other material. **Occurrence:** occurrenceID: 08849EC4-519D-5C87-9FBF-9FFC944593A6; **Location:** higherGeography: Asia; country: China; countryCode: CH; verbatimLocality: Tacheng, Weixi, YN; decimalLatitude: 27.5000000; decimalLongitude: 98.9600000; **Identification:** identificationReferences: Liu et al., 2012, 2018; identificationRemarks: getotyped
- **Type status:** Other material. **Occurrence:** occurrenceID: 6AD3F10C-389F-5400-8404-103260E7C739; **Location:** higherGeography: Asia; country: China; countryCode: CH; verbatimLocality: Xinshengqiao, Lanping, YN; decimalLatitude: 26.4800000; decimalLongitude: 99.3200000; **Identification:** identificationReferences: Liu et al., 2012, 2018; identificationRemarks: getotyped
- **Type status:** Other material. **Occurrence:** occurrenceID: 1F9527EB-E63A-5E4A-89E1-B6EBAF10DF0E; **Location:** higherGeography: Asia; country: China; countryCode: CH; verbatimLocality: Nuodeng, Yunlong, YN; decimalLatitude: 25.8900000; decimalLongitude: 99.367; **Identification:** identificationReferences: Liu et al., 2012, 2018; identificationRemarks: getotyped
- **Type status:** Other material. **Occurrence:** occurrenceID: E01304B1-D5D7-5CE9-BBB2-DEC894BC6BDE; **Location:** higherGeography: Asia; country: China; countryCode: CH; verbatimLocality: Caojian, Yunlong, YN; decimalLatitude: 25.6600000; decimalLongitude: 99.1320000; **Identification:** identificationReferences: Liu et al., 2012, 2018; identificationRemarks: getotyped
- **Type status:** Other material. **Occurrence:** occurrenceID: F417F8C9-C45B-52F9-B8EC-250A15AD86FE; **Location:** higherGeography: Asia; country: China; countryCode: CH; verbatimLocality: Xujiaba, Mt. Ailao, YN; decimalLatitude: 23.2430000; decimalLongitude: 102.3700000; **Identification:** identificationReferences: Liu et al., 2012, 2018; identificationRemarks: getotyped
- **Type status:** Other material. **Occurrence:** occurrenceID: 134B1379-3191-578F-ABA4-5A6F7CF565A4; **Location:** higherGeography: Asia; country: China; countryCode: CH; verbatimLocality: Shale, Nanjian, YN; decimalLatitude: 24.9100000; decimalLongitude: 100.4100000; **Identification:** identificationReferences: Liu et al., 2012, 2018; identificationRemarks: getotyped
- **Type status:** Other material. **Occurrence:** occurrenceID: 96EFA602-5E72-5EF0-823D-57BC22C4605A; **Location:** higherGeography: Asia; country: China; countryCode: CH; verbatimLocality: Baohua, Nanjian, YN; decimalLatitude: 24.9110000; decimalLongitude: 100.4910000; **Identification:** identificationReferences: Liu et al., 2012, 2018; identificationRemarks: getotyped
- **Type status:** Other material. **Occurrence:** occurrenceID: 49AF39F1-2E94-50CF-9B27-91F861637981; **Location:** higherGeography: Asia; country: China; countryCode: CH; verbatimLocality: Modaohe, Jindong, YN; decimalLatitude: 23.3500000; decimalLongitude: 101.5000000; **Identification:** identificationReferences: Liu et al., 2012, 2018; identificationRemarks: getotyped
- **Type status:** Other material. **Occurrence:** occurrenceID: 8765AB0D-0FDC-503E-902A-C661E72FD871; **Location:** higherGeography: Asia; country: China; countryCode: CH; verbatimLocality: Wangjiajing, Jindong, YN; decimalLatitude: 24.4100000; decimalLongitude: 100.7800000; **Identification:** identificationReferences: Liu et al., 2012, 2018; identificationRemarks: getotyped
- **Type status:** Other material. **Occurrence:** occurrenceID: 909B5F01-202A-5734-9A37-F4109F45545F; **Location:** higherGeography: Asia; country: China; countryCode: CH; verbatimLocality: Raomalu, Jindong, YN; decimalLatitude: 24.0200000; decimalLongitude: 101.0000000; **Identification:** identificationReferences: Liu et al., 2012, 2018; identificationRemarks: getotyped
- **Type status:** Other material. **Occurrence:** occurrenceID: 69C99398-7BAF-59DD-AA98-8602D0A2FD3B; **Location:** higherGeography: Asia; country: China; countryCode: CH; verbatimLocality: Dazhaizi, Jindong, YN; decimalLatitude: 24.2200000; decimalLongitude: 100.8800000; **Identification:** identificationReferences: Liu et al., 2012, 2018; identificationRemarks: getotyped
- **Type status:** Other material. **Occurrence:** occurrenceID: 1AC07AD9-3632-50EE-8E28-2040780F735E; **Location:** higherGeography: Asia; country: China; countryCode: CH; verbatimLocality: Huangcaoling, Jindong, YN; decimalLatitude: 24.2000000; decimalLongitude: 101.1600000; **Identification:** identificationReferences: Liu et al., 2012, 2018; identificationRemarks: getotyped
- **Type status:** Other material. **Occurrence:** occurrenceID: 404EB792-57D2-5D2F-B00B-7673C27BB4AB; **Location:** higherGeography: Asia; country: China; countryCode: CH; verbatimLocality: Luodang, Fengqing, YN; decimalLatitude: 24.5270000; decimalLongitude: 100.0200000; **Identification:** identificationReferences: Liu et al., 2012, 2018; identificationRemarks: getotyped
- **Type status:** Other material. **Occurrence:** occurrenceID: 7A75918C-022C-5CD4-9BA3-92E2A9139F73; **Location:** higherGeography: Asia; country: China; countryCode: CH; verbatimLocality: Lushi, Fengqing, YN; decimalLatitude: 24.8430000; decimalLongitude: 100.0040000; **Identification:** identificationReferences: Liu et al., 2012, 2018; identificationRemarks: getotyped
- **Type status:** Other material. **Occurrence:** occurrenceID: 9D9CD1AB-05C7-5373-BA26-2EA60534B822; **Location:** higherGeography: Asia; country: China; countryCode: CH; verbatimLocality: Wumeng, Luquan, YN; decimalLatitude: 26.0210000; decimalLongitude: 102.7900000; **Identification:** identificationReferences: Liu et al., 2012, 2018; identificationRemarks: getotyped
- **Type status:** Other material. **Occurrence:** occurrenceID: 06C87838-1AF9-553A-AFBF-A0FE72375826; **Location:** higherGeography: Asia; country: China; countryCode: CH; verbatimLocality: Fenghuangchang, Mt. Jiaozi, YN; decimalLatitude: 25.9500000; decimalLongitude: 102.7000000; **Identification:** identificationReferences: Liu et al., 2012, 2018; identificationRemarks: getotyped
- **Type status:** Other material. **Occurrence:** occurrenceID: 1CF57BD5-FE70-5391-9754-AADB3DA6637D; **Location:** higherGeography: Asia; country: China; countryCode: CH; verbatimLocality: Hongtudi, Mt. Jiaozi, YN; decimalLatitude: 26.1000000; decimalLongitude: 102.5300000; **Identification:** identificationReferences: Liu et al., 2012, 2018; identificationRemarks: getotyped
- **Type status:** Other material. **Occurrence:** occurrenceID: BF44FABD-ADDA-582A-A8AF-3955A7833814; **Location:** higherGeography: Asia; country: China; countryCode: CH; verbatimLocality: Daohe, Yongde, YN; decimalLatitude: 24.2440000; decimalLongitude: 99.6320000; **Identification:** identificationReferences: Liu et al., 2012, 2018; identificationRemarks: getotyped
- **Type status:** Other material. **Occurrence:** occurrenceID: 655A12A3-C87A-5B46-A284-923F2376350F; **Location:** higherGeography: Asia; country: China; countryCode: CH; verbatimLocality: Zhongjiaochang, Yongde, YN; decimalLatitude: 23.9900000; decimalLongitude: 99.5900000; **Identification:** identificationReferences: Liu et al., 2012, 2018; identificationRemarks: getotyped
- **Type status:** Other material. **Occurrence:** occurrenceID: 8995A5E7-9F86-52CA-8279-0BCBB5AE382C; **Location:** higherGeography: Asia; country: China; countryCode: CH; verbatimLocality: Taojin river, Yongde, YN; decimalLatitude: 23.8700000; decimalLongitude: 99.4900000; **Identification:** identificationReferences: Liu et al., 2012, 2018; identificationRemarks: getotyped
- **Type status:** Other material. **Occurrence:** occurrenceID: AE0A5CE8-870C-50A5-996A-F6797FD1B85A; **Location:** higherGeography: Asia; country: China; countryCode: CH; verbatimLocality: Yangwanshun, Yongde, YN; decimalLatitude: 24.0600000; decimalLongitude: 99.6140000; **Identification:** identificationReferences: Liu et al., 2012, 2018; identificationRemarks: getotyped
- **Type status:** Other material. **Occurrence:** occurrenceID: 1A5F18A3-08AA-57E8-A8E7-B5E2D6B010E5; **Location:** higherGeography: Asia; country: China; countryCode: CH; verbatimLocality: Xiaoxueshan, Yongde, YN; decimalLatitude: 24.0580000; decimalLongitude: 99.6130000; **Identification:** identificationReferences: Liu et al., 2012, 2018; identificationRemarks: getotyped
- **Type status:** Other material. **Occurrence:** occurrenceID: 8BC2B1CD-12F1-54AD-932B-A64635A956AB; **Location:** higherGeography: Asia; country: China; countryCode: CH; verbatimLocality: Yinchangjie,Yongde, YN; decimalLatitude: 24.1950000; decimalLongitude: 99.6580000; **Identification:** identificationReferences: Liu et al., 2012, 2018; identificationRemarks: getotyped
- **Type status:** Other material. **Occurrence:** occurrenceID: BF856341-A9FE-5318-80F6-5354C6DBAE1B; **Location:** higherGeography: Asia; country: China; countryCode: CH; verbatimLocality: Ganhe, Yongde, YN; decimalLatitude: 23.8710000; decimalLongitude: 99.2730000; **Identification:** identificationReferences: Liu et al., 2012, 2018; identificationRemarks: getotyped
- **Type status:** Other material. **Occurrence:** occurrenceID: 7A6BCEA9-066B-51CC-A08F-05AE43D14305; **Location:** higherGeography: Asia; country: China; countryCode: CH; verbatimLocality: Bangdong, Lincang, YN; decimalLatitude: 24.2830000; decimalLongitude: 100.4290000; **Identification:** identificationReferences: Liu et al., 2012, 2018; identificationRemarks: getotyped
- **Type status:** Other material. **Occurrence:** occurrenceID: 36F7959D-D1A8-52B4-AE02-E397C97B8606; **Location:** higherGeography: Asia; country: China; countryCode: CH; verbatimLocality: Mengku, Mt. Bangma, YN; decimalLatitude: 23.5350000; decimalLongitude: 99.8000000; **Identification:** identificationReferences: Liu et al., 2012, 2018; identificationRemarks: getotyped
- **Type status:** Other material. **Occurrence:** occurrenceID: D40B3BBB-967B-56A7-B858-4BE1DC80CCAB; **Location:** higherGeography: Asia; country: China; countryCode: CH; verbatimLocality: Xiaobangma, Mt. Bangma, YN; decimalLatitude: 23.4600000; decimalLongitude: 99.7280000; **Identification:** identificationReferences: Liu et al., 2012, 2018; identificationRemarks: getotyped
- **Type status:** Other material. **Occurrence:** occurrenceID: B0181B51-07B0-5806-9310-1467F6F603A5; **Location:** higherGeography: Asia; country: China; countryCode: CH; verbatimLocality: Dabangma, Mt. Bangma, YN; decimalLatitude: 23.5450000; decimalLongitude: 99.7000000; **Identification:** identificationReferences: Liu et al., 2012, 2018; identificationRemarks: getotyped
- **Type status:** Other material. **Occurrence:** occurrenceID: 49047A90-213E-525C-ADBC-AC9802E0A14B; **Location:** higherGeography: Asia; country: China; countryCode: CH; verbatimLocality: Dizhengdang, Gongshan, YN; decimalLatitude: 28.0790000; decimalLongitude: 98.3260000; **Identification:** identificationReferences: Liu et al., 2012, 2018; identificationRemarks: getotyped
- **Type status:** Other material. **Occurrence:** occurrenceID: D6C72930-95EB-56CC-A5D9-B64C6FD2CEC9; **Location:** higherGeography: Asia; country: China; countryCode: CH; verbatimLocality: Baihualing, Baoshan, YN; decimalLatitude: 25.1300000; decimalLongitude: 98.7700000; **Identification:** identificationReferences: Liu et al., 2012, 2018; identificationRemarks: getotyped
- **Type status:** Other material. **Occurrence:** occurrenceID: 16281402-97D6-57BD-8150-11E7E611A7D4; **Location:** higherGeography: Asia; country: China; countryCode: CH; verbatimLocality: Pianma, Lushui, YN; decimalLatitude: 26.0090000; decimalLongitude: 98.6370000; **Identification:** identificationReferences: Liu et al., 2012, 2018; identificationRemarks: getotyped
- **Type status:** Other material. **Occurrence:** occurrenceID: EFDBF40B-219C-5A07-92CC-F4ED8D2DE23C; **Location:** higherGeography: Asia; country: China; countryCode: CH; verbatimLocality: Yaojiaping, Tengchong, YN; decimalLatitude: 26.9100000; decimalLongitude: 98.7900000; **Identification:** identificationReferences: Liu et al., 2012, 2018; identificationRemarks: getotyped
- **Type status:** Other material. **Occurrence:** occurrenceID: 36D4A58B-40B9-53AF-A324-5219E06799DF; **Location:** higherGeography: Asia; country: China; countryCode: CH; verbatimLocality: Shibali, Mt. Gaoligong, YN; decimalLatitude: 25.1300000; decimalLongitude: 98.7160000; **Identification:** identificationReferences: Liu et al., 2012, 2018; identificationRemarks: getotyped
- **Type status:** Other material. **Occurrence:** occurrenceID: F34BC05D-FDFD-5CFA-947E-3ECBA43898A0; **Location:** higherGeography: Asia; country: China; countryCode: CH; verbatimLocality: Mingguang, Tengcheng, YN; decimalLatitude: 25.7260000; decimalLongitude: 98.5560000; **Identification:** identificationReferences: Liu et al., 2012, 2018; identificationRemarks: getotyped
- **Type status:** Other material. **Occurrence:** occurrenceID: CCC7709D-C6B7-5891-BEB4-68FDA1511419; **Location:** higherGeography: Asia; country: China; countryCode: CH; verbatimLocality: Liangshan, Longlin, YN; decimalLatitude: 24.3500000; decimalLongitude: 98.8000000; **Identification:** identificationReferences: Liu et al., 2012, 2018; identificationRemarks: getotyped
- **Type status:** Other material. **Occurrence:** occurrenceID: 5C57F079-EDD3-5198-AD94-EAD0FA96BA77; **Location:** higherGeography: Asia; country: China; countryCode: CH; verbatimLocality: Kunming, YN; decimalLatitude: 24.9200000; decimalLongitude: 102.8100000; **Identification:** identificationReferences: Liu et al., 2012, 2018; identificationRemarks: getotyped
- **Type status:** Other material. **Occurrence:** occurrenceID: 81C005AC-44E0-5573-BC72-AFA5E5EEB425; **Location:** higherGeography: Asia; country: China; countryCode: CH; verbatimLocality: Mt. Wuliang, YN; decimalLatitude: 24.8420000; decimalLongitude: 100.0000000; **Identification:** identificationReferences: Liu et al., 2012, 2018; identificationRemarks: getotyped
- **Type status:** Other material. **Occurrence:** occurrenceID: 3BE7BF2A-9D5E-514E-A2F9-FBDBFC863CFB; **Location:** higherGeography: Asia; country: China; countryCode: CH; verbatimLocality: Nyingchi County, Tibet Autonomous Region; decimalLatitude: 29.6100000; decimalLongitude: 94.3600000; **Identification:** identificationReferences: Ge et al., 2019; identificationRemarks: getotyped
- **Type status:** Other material. **Occurrence:** occurrenceID: 531D4AB7-84AB-5518-8CC7-91B5BB07494E; **Location:** higherGeography: Asia; country: China; countryCode: CH; verbatimLocality: Milin County, Tibet Autonomous Region; decimalLatitude: 29.2000000; decimalLongitude: 94.2100000; **Identification:** identificationReferences: Ge et al., 2019; identificationRemarks: getotyped
- **Type status:** Other material. **Occurrence:** occurrenceID: C01C1A57-A690-5C1B-99D2-BDE5417943B8; **Location:** higherGeography: Asia; country: China; countryCode: CH; verbatimLocality: Sejila Mountain, Tibet Autonomous Region; decimalLatitude: 30.0000000; decimalLongitude: 94.9500000; **Identification:** identificationReferences: Ge et al., 2019; identificationRemarks: getotyped
- **Type status:** Other material. **Occurrence:** occurrenceID: 20E79EE3-B28C-563B-BBB6-F52C736D5231; **Location:** higherGeography: Asia; country: China; countryCode: CH; verbatimLocality: Ailao Mountain, Jingdong County, Yunnan Province; decimalLatitude: 24.2150000; decimalLongitude: 101.3270000; **Identification:** identificationReferences: Ge et al., 2019; identificationRemarks: getotyped
- **Type status:** Other material. **Occurrence:** occurrenceID: 87936155-54C8-5A9F-B1EE-9505FF122283; **Location:** higherGeography: Asia; country: China; countryCode: CH; verbatimLocality: Daxue Mountain, Lincang City, Yunnan Province; decimalLatitude: 24.1030000; decimalLongitude: 99.6400000; **Identification:** identificationReferences: Ge et al., 2019; identificationRemarks: getotyped
- **Type status:** Other material. **Occurrence:** occurrenceID: 3A4EE7EC-9D6B-5F3D-9285-58FDFF56831D; **Location:** higherGeography: Asia; country: China; countryCode: CH; verbatimLocality: Shangri-La County, Yunnan Province; decimalLatitude: 27.8000000; decimalLongitude: 99.7000000; **Identification:** identificationReferences: Ge et al., 2019; identificationRemarks: getotyped
- **Type status:** Other material. **Occurrence:** occurrenceID: DFE360D0-30EB-5294-84E9-16283BE513B4; **Location:** higherGeography: Asia; country: China; countryCode: CH; verbatimLocality: Yulong County, Yunnan Province; decimalLatitude: 26.8000000; decimalLongitude: 100.0000000; **Identification:** identificationReferences: Ge et al., 2019; identificationRemarks: getotyped
- **Type status:** Other material. **Occurrence:** occurrenceID: CFFA849A-0976-514A-9701-63E79F37E8CA; **Location:** higherGeography: Asia; country: Vietnam; countryCode: VN; verbatimLocality: Ta Xua NR, xa Xim Vang, ban Suong Chong; decimalLatitude: 21.3199640; decimalLongitude: 104.4080680; **Identification:** identificationReferences: Balakirev et al. 2025; identificationRemarks: getotyped

#### Diagnosis

The tooth morphology is similar to that of *Apodemus draco*, with three cusps on the lingual side of the third upper molar, with the posterior-lingual cusp (t7). The hair is rather long, usually brown, sometimes with a distinct black area on the back. Some individuals have a black area in the center of the entire back; the tail is slightly longer than the body length, about 105-110% of the body length. The ears are large, about 18mm, with black ear edges, or the entire front of the ear is blackish, and the very short hairs. The back of the ear is also black, with short hair. The forehead is usually gray-black. The boundary between the back and abdomen is obvious. The base of the hair on the abdomen is grey, and the tip of the hair is gray-white. The tail is two-colored above and below, gray-black on the back and gray-white on the ventral side. About 50% of individuals have a hair tuft at the end of the tail. The back of the front and back feet are pure white. The claws are milky white, and both the front and back feet have 5 fingers (Smith and Xie 2008, Liu et al. 2018, Liu et al. 2012).

#### Distribution

The species is widespread in southwest China, including Yunnan and eastern Tibet, as well as the Gongbu area in southern Tibet. Also distributed in the Three Parallel Rivers region in western China, including Gaoligong Mountain, Meili Snow Mountain, Baima Snow Mountain. Probably may also be found in Myanmar and northern India (Liu et al. 2012, Liu et al. 2017, Liu et al. 2018, Liu et al. 2004).

#### Ecology

Inhabits the mountain forests and shrubs at Mid-Higher elevation belt. It prefers humid areas and sometimes lives in caves. Feeds on a variety of plant seeds and fruits, and also eats green parts of plants and some insects. Active mainly at night and dawn. The breeding period is from April to November. Each litter has at least 3 pups, up to 10 pups, and an average of 5-7 pups (Xia 1984, Yang et al. 2002, Smith and Xie 2008).

#### Conservation

IUCN LC (as treated the same as *A. draco*).

#### Notes

First records of *Apodemus ilex* in Vietnam.

## Analysis

**Figure 2.**
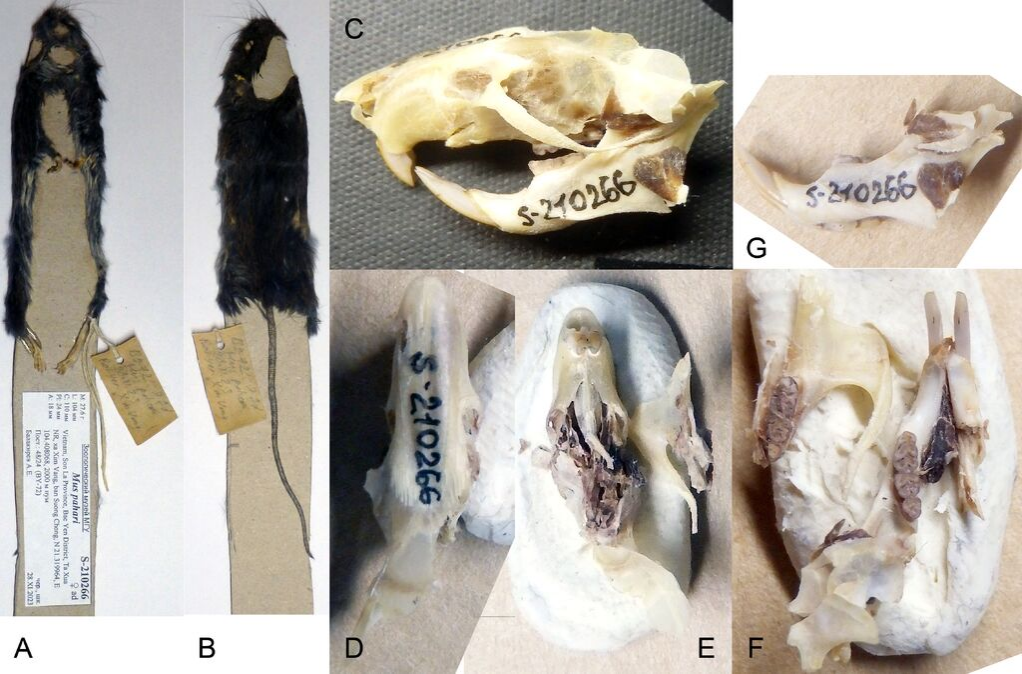
*Apodemus ilex* sample from Vietnam (Son La Province, Bac Yen District, Xin Vang commune, near village Suong Chong, Ta Xua Nature Reserve, 21.319964N; 104.408068E), field label BY-72, ZMMU S-210266. **A** Flat skin ventral view; **B** Flat skin dorsal view; **C** Broken skull, assembled; **D** Rostral part of skull, dorsal view; **E** Rostral part of skull ventral view; **F** Left lower jaw and part of left orbital skull zone with labial teeth; **G** Lower jaw, side view.

During the captures, among dozens of individuals of other species, including new to Vietnam (Balakirev et al. 2025) and new to science mammal species described recently (Balakirev et al. 2024), a specimen of a small mouse was captured, which unexpectedly appeared to be a representative of the genus *Apodemus*. This adult female was caught by a snap trap at Xin Vang commune, near village Suong Chong, Ta Xua Nature Reserve, 21.319964N; 104.408068E, about 2000 m a.s.l. The animal's skull was severely damaged by the trap impact. The pelt was wet from rain, and this specimen was initially misidentified visually as *Mus pahari*. These mouse species are very similar in colouration and external body size and proportions, so they can be easily confused without analyzing their dental structure. Later, after processing the skulls and skins and transferring them to the museum collection, it became possible to conduct a detailed analysis of the cheek tooth structure, which allowed us to confidently confirm the discovery of a representative of the genus *Apodemus*. This specimen, originally registered under field number BT-72, is currently held in the ZMMU collection under museum number S-210266 and, apparently, represents the first record of *Apodemus* in Vietnam Fig. 2. The animal was caught in a mountain forest at an altitude of approximately 2,000 m above sea level. Near the tree line, the trap was set on the ground, using fresh peanuts as bait.

On the skull, the characteristic features of the genus *Apodemus* are clearly visible in the structure of the chewing surfaces of the molars, namely, the first upper molar does not exceed half the length of the molar tooth row and is present with the posterior-lingual cusp (t7). This pattern, together with the absence of a characteristic cusp on the inner surface of the upper incisors, is a perceptible difference between wood and field mice *Apodemus*/*Sylvaemus* and common house mice of the genus *Mus* (Smith and Xie 2008). Unfortunately, the skull of the animal was heavily damaged and broken into pieces, which does not allow us to provide complete dimensional characteristics of the skull here; however, the measurements of the upper and lower rows of molars are quite consistent with the species characteristics for *A. draco* and *A. ilex*. For our specimen BY-72, the length of the upper tooth row was M^1-3^ – 4.06 mm; lower tooth row m_1-3_ – 4.02 mm; upper molar M^1^ length – 1.97 mm; upper molar M^1^ width – 1.28 mm; lower molar m_1_ length – 1.897 mm; lower molar m_1_ width – 1.11. The overall dimensions of the animal corresponded to or even slightly exceeded the dimensions of *Apodemus* species characteristic of Yunnan, China. Head and Body length – 104 mm; Tail length – 110 mm.; Hind feet length (with claws) – 24 mm.; Ear loop length – 18 mm.; Weight – 27.6 g. It should also be noted that the fur colour of our specimen is very dark and is more similar to that characteristic of *A. latronum* (Smith and Xie 2008). Dorsal pelage is dark brown, ventral pelage is grayish white, indistinctly demarcated from the dorsal pelage. Tail approximately equal to head and body length, dark brown above, paler below. Ears dark blackish brown, as dark as the surrounding parts of the head and shoulders.

Obviously, due to the great morphological similarity and high geographic variability of Chinese *Apodemus* species, a precise species attribution should have been provided by the genetic analysis we conducted based on the full-length cytochrome *b* gene. The use of the BLAST procedure unconditionally showed that the studied sample undoubtedly belongs to *Apodemus ilex*. The closest related nucleotide sequences available in the GenBank database (as of November 1, 2025) turned out to be haplotypes 13, 71, and 128 of this species (IDs JF503210, JF503140b, JF503142) originating from central Yunnan (Liu et al., 2012) with a sequence identity level of 97.63% (*d* T2P = 0.0237).

For comparative phylogenetic analysis, we collected all available *Apodemus ilex* sequences with known geographic locations from the GenBank database, a total of 214 nucleotide sequences from 74 geographic locations, including our original sample from Vietnam (Suppl. material 1 and Taxon Treatment section table) previously used in the survey of Liu et al. (2004), Liu et al. (2012), Liu et al. (2017), Liu et al. (2018), Ge et al. (2019), Song et al. (2021) and several unpublished sequences from the GenBank public domain. Due to the limitations of the original material, the aim of our survey did not include a full phylogenetic analysis of the mitochondrial genetic lineages of *Apodemus ilex*. To assess the position of the newly discovered sample, we selected 18 sequences (GeneBank IDs JF503110, JF503140, JF503142, JF503147, JF503150, JF503155, JF503157, JF503166, JF503169, JF503175, JF503194, JF503195, JF503196, JF503201, JF503202, JF503217, JF503219, JF503233), representing the main genetic clades known in this species. Two *Apodemus draco* specimens (KP694301, HQ333255) from Sichuan Province, China, were used as outgroups.

Phylogenetic analysis using the minimal evolution (ME) method revealed that the Vietnamese sample belongs to the E1 (Eastern) phylogenetic group, according to the terminology used by Liu et al. (2012) Fig. 3. Our samples form another independent phylogenetic clade within the Eastern phylogenetic group. This point can be assessed more precisely as more material accumulates. At the same time, it should be noted that the obvious genetic uniqueness of the sample obviously rejects the version of its accidental introduction into the territory of Vietnam and indicates the presence of a unique natural population.

**Figure 4.**
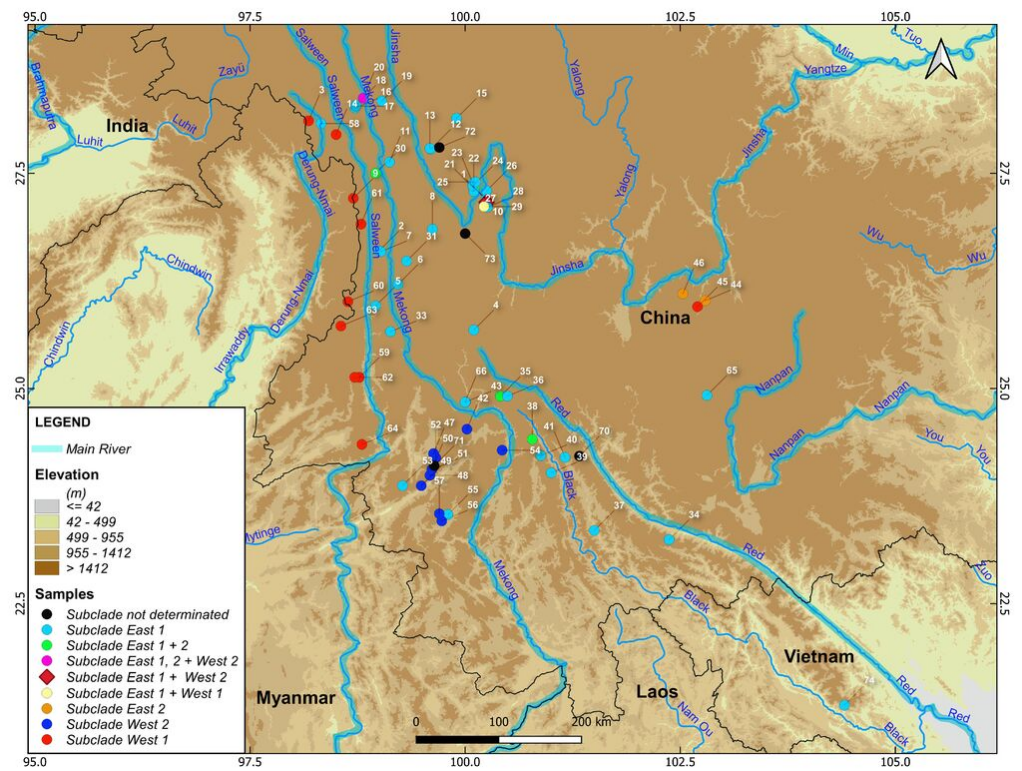
Genetically dated distribution range of *Apodemus ilex* and its phylogenetic structure (see Liu et al. 2012; Ge et al., 2017 and Supplement table 1 for details and coordinates). The new locality of the species in Vietnam, indicated as location number 74, is the most southerly for this species and the genus as a whole.

Phylogroup E is widely distributed in the southern Hengduan Mountains and the Yunnan-Guizhou Plateau, as well as in the upper reaches of the Mekong, Hong Ha, and Da Rivers (Fig. 4). As can be seen, the new occurrence point significantly expands the known species range to the south. It is more than 230 kilometres away from the previously documented southernmost locality (point 34, Mount Xujiaba, Ailao, Yunnan, 23.243N 102.370E) along the shortest distance.

## Discussion

Many patterns in the geographic distribution of small mammals can be explained based on the refugial model of species evolution. The refugia hypothesis states that animal populations are reduced when available habitats decrease during cold episodes in the Pleistocene climatic oscillation (Heaney 1986, Cracraft and Prum 2017) and repeatedly increase with considerable expansion during warm interglacials. In Indochina, reduction and expansion of forests, especially mountain forests, could have affected phylogeographic patterns in many species of mammals (Heuertz et al. 2010, Latinne et al. 2015, Stefenon et al. 2019). The possibilities of range expansion, in turn, are determined primarily by the location of barriers and natural refugia.

The discovery point of our new find lies at the southernmost end of the Hoang Lien Mountains. These mountains are a direct continuation of the Chinese Hengduan, the most important refugial region in China, and the Yunnan Plateau. Hoang Lien Son Mountain range lies in the provinces of Lao Cai, Yen Bai, Lai Chau, and Son La in northwest Vietnam. They are one of the most biologically diverse landscapes in Vietnam and the wider Indochinese region. The Mountain Range, with an altitudinal range from 300 to 3,143 meters, lies at the junction of two biogeographic realms (Palearctic and Indo-Malayan). Based on the natural differentiation of climatic factors, Vũ Tự Lập (1976) recognized in Vietnam vegetation three mountain elevation zones: from 0 to 600 m (including three sub-belts), 600–2,600 m (including three sub-belts), and over 2,600 m. All these three biomes (tropical dry forests/woodlands, tropical humid forests, and subtropical/temperate rainforest/woodlands) are distinguished simultaneously only within the Hoang Lien Son range. This makes this range an ideal ecological corridor for the dispersal routes of forest species, both plants and animals. Flora diversity in Hoang Lien Nature Park (Lao Cai province) is treated as class A — the highest biodiversity value of Vietnam by the Global Environment Fund. And in all respects, it is similar to the rich flora of Yunnan. (Wang 1939, Cao and H 1997, Zheng et al. 2006a, Zheng et al. 2006b, Zhu et al. 2006). This circumstance should also be reflected in the structure of animal communities living in these ecosystems and subsisting on their resources (Wang and Jin 1987).

There have been numerous studies evaluating climate differentiation as well as ecological surveys and vegetation distribution investigations associated with elevation belts (Vo 1970, Kem and Dilger 1994, Ly et al. 1996, Thin and Harder 1996, Pham 1999, Vu and Nguyen 2005). The flora screening within and around Hoang Lien Nature Park revealed the presence of a total of 3252 species, 1121 genera, 230 families of 6 vascular plants (see Tri 2009, Yen et al. 2019 for details). At the belt between 1700–2200 m, where *Apodemus ilex* was discovered, there are recorded 5 phyla (83.33%), 165 families (71.74%), 521 genera and 1115 species of plants. That is, in terms of flora, and consequently in terms of habitats for fauna, this region is in all respects similar to the mountain systems of southern China (Vu and Nguyen 2005). The same plant communities are also usually distributed far to the south in Son La Province, up to the very end of the mountain ridge where Ta Xua Nature Reserve is situated. From this perspective, the distribution and spread of temperate rodent species characteristic of the mountain forests of southern China does not seem surprising, but rather predictable. The only surprising thing is that they had not been discovered here by numerous researchers earlier.

Paleoclimatic change has also shaped the genetic structure of many species. The glacial-interglacial cycles not only resulted in inter- and intraspecific divergence but also may have led to population expansions. The haplotype we discovered is located near the root of the phylogenetic structure of the major E1 clade identified by Liu et al. (2012), which estimates the age of the divergence between the eastern and western populations of *A. ilex* (0.62 Mya) was within the Yulong glaciation (0.73–0.5 Mya) (Wu et al. 2001) and the simultaneous divergences of the subclades E1/E2 (0.33 Mya) and W1/W2 (0.32 Mya) were consistent with the Lijiang glaciation (0.31–0.13 Mya) (Zhao et al. 2007) at the Hengduan Mountains. Equal divergence rates suggest that the Vietnamese phylogroup is coeval with the Chinese ones, and its core position indicates that this population is likely not the result of accidental introduction or a recent expansion but also has a refugial origin, likely associated with the Hoang Lien Son Mountain range. If the divergence timing is correct, they provide evidence that "nunatak refugia" existed in these mountains. Nunataks are refugia in mountain ranges above the glaciers and are snow-free during glaciations (Holderegger and Thiel‐Egenter 2009). Accordingly, *A. ilex* probably widely colonized the southern Hengduan Mountains before the Yulong glaciation. The development of valley glaciers during the recent Yulong and Lijiang glaciation resulted in geographical isolation, but the populations survived in multiple refugia (Bettin et al. 2007, Holderegger and Thiel‐Egenter 2009), leading to the diversification of the clades/subclades. This isolation in the same refugia undoubtedly affected populations of other sympatric species of rodents and speciation processes within them, particularly species of the genera *Typhlomys* and *Eothenomys*, also associated with mountain ecosystems (Balakirev et al. 2025, Balakirev et al. 2024).

Due to the obvious limitations of data available, it is currently difficult to determine whether the range of *Apodemus ilex* in Vietnam is continuous or has disjunction. The former is more likely, as there are no significant ecological barriers to the distribution of small mammals along the Hoang Lien Son Ridge, and the altitudinal vegetation belts are generally continuous on both slopes. Therefore, this species may have been found in other areas of Vietnam, including lowlands along the Da “Black” and Hong “Red” River valleys, where field mice could find a suitable habitat. We hope that additional research in the country's northeastern provinces will soon clarify this issue. As for its conservation status, given the species' significant range in China, it is unlikely to be classified as endangered, although it should obviously still be considered rare and data-deficient in Vietnam.

## Supplementary Material

XML Treatment for Apodemus (Apodemus) ilex

07256B0A-3116-51CB-AB2F-6FF3CC389837 10.3897/BDJ.14.e179239.suppl1 Supplementary material 1 Cyt *b* gene Data type genetic Brief description Cyt *b* gene of *Apodemus ilex* alignment. File: oo_1468185.gb https://binary.pensoft.net/file/1468185 Alexander E. Balakirev

## Figures and Tables

**Figure 3.**
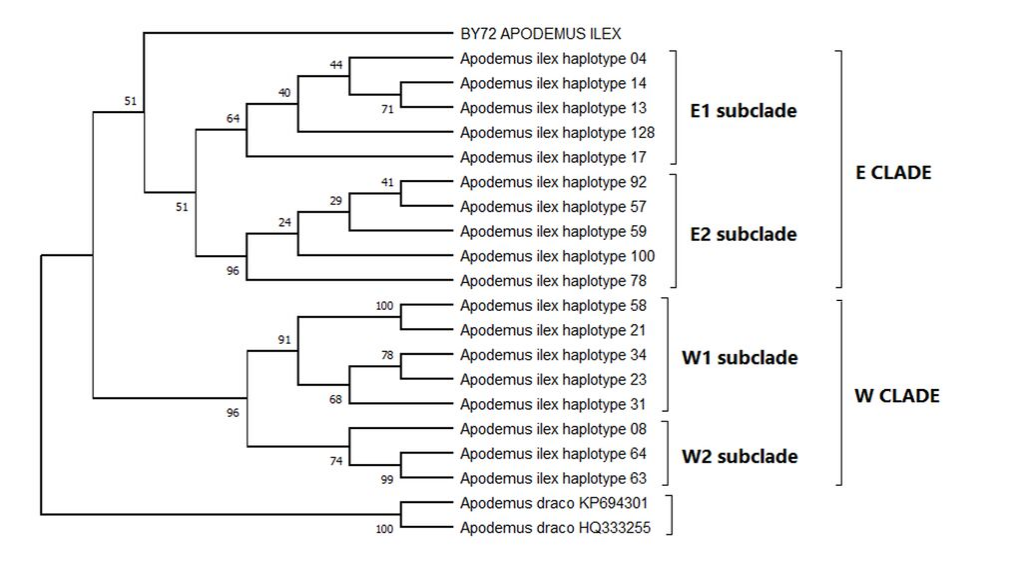
Phylogenetic tree (ME, *cyt b*, 1140 bp) illustrating the position of sample BY-72 in the phylogenetic structure of *Apodemus ilex*. Bootstrap values are indicated above the nodes.
